# 3,6-Diaza­octane-1,8-diaminium diiodide

**DOI:** 10.1107/S1600536812030127

**Published:** 2012-07-07

**Authors:** Ignacy Cukrowski, Adedapo S. Adeyinka, David C. Liles

**Affiliations:** a Department of Chemistry, University of Pretoria, Private Bag X20, Hatfield 0028, South Africa

## Abstract

The asymmetric unit of the title salt, C_6_H_20_N_4_^2+^·2I^−^, comprises half a 3,6-diaza­octane-1,8-diaminium dication plus an I^−^ anion. The dications are symmetrical and lie across crystallographic centres of inversion. In the crystal, the ions form a network involving mainly weak N—H⋯I inter­molecular inter­actions: two H atoms of the ammonium group form inter­actions with two I^−^ anions and the H atom of the secondary amine forms a weak inter­action with a third I^−^ cation. The third ammonium H atom is hydrogen bonded to a secondary amine of an adjacent cation. The backbone of the cation does not form a uniformly *trans* chain, but is ‘kinked’ [C—N—C—C torsion angle = 71.5 (2)°], probably to accommodate the direct hydrogen bond between the ammonium group and the secondary amine in an adjacent cation.

## Related literature

For the structure of a dihydrate of the title compound, together with its isostructural Cl^−^ and Br^−^ analogues, see Ilioudis *et al.* (2000 ▶).
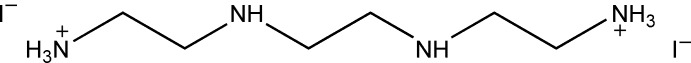


## Experimental

### 

#### Crystal data

C_6_H_20_N_4_^2+^·2I^−^
*M* *_r_* = 402.06
Orthorhombic, 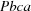

*a* = 8.1253 (2) Å
*b* = 8.6138 (2) Å
*c* = 18.9368 (4) Å
*V* = 1325.38 (5) Å^3^
*Z* = 4
Mo *K*α radiation
μ = 4.71 mm^−1^
*T* = 180 K
0.14 × 0.10 × 0.07 mm

#### Data collection

Nonius KappaCCD diffractometer
Absorption correction: multi-scan (*SORTAV*; Blessing, 1995 ▶) *T*_min_ = 0.688, *T*_max_ = 0.820
13267 measured reflections
2635 independent reflections
1905 reflections with *I* > 2σ(*I*)
*R*_int_ = 0.042

#### Refinement

*R*[*F* ^2^ > 2σ(*F* ^2^)] = 0.023
*wR*(*F* ^2^) = 0.053
*S* = 0.97
2635 reflections
67 parameters
1 restraint
H atoms treated by a mixture of independent and constrained refinement
Δρ_max_ = 0.55 e Å^−3^
Δρ_min_ = −1.05 e Å^−3^

### 

Data collection: *COLLECT* (Nonius, 1998 ▶); cell refinement: *SCALEPACK* (Otwinowski & Minor, 1997 ▶); data reduction: *DENZO* (Otwinowski & Minor, 1997 ▶), *SCALEPACK* and *SORTAV* (Blessing, 1995 ▶); program(s) used to solve structure: *SIR92* (Altomare *et al.*, 1994 ▶); program(s) used to refine structure: *SHELXL97* (Sheldrick, 2008 ▶); molecular graphics: *ORTEP-3 for Windows* (Farrugia, 1997 ▶), *POV-RAY* (Cason, 2004 ▶) and *Mercury* (Macrae *et al.*, 2008 ▶); software used to prepare material for publication: *SHELXL97* and *PLATON* (Spek, 2009 ▶).

## Supplementary Material

Crystal structure: contains datablock(s) I, global. DOI: 10.1107/S1600536812030127/jj2132sup1.cif

Structure factors: contains datablock(s) I. DOI: 10.1107/S1600536812030127/jj2132Isup2.hkl

Supplementary material file. DOI: 10.1107/S1600536812030127/jj2132Isup3.cml

Additional supplementary materials:  crystallographic information; 3D view; checkCIF report

## Figures and Tables

**Table 1:** Hydrogen-bond geometry (Å, °)

*D*—H⋯*A*	*D*—H	H⋯*A*	*D*⋯*A*	*D*—H⋯*A*
N1—H1*C*⋯N4^i^	1.10 (2)	1.71 (2)	2.800 (2)	168.3 (19)
N1—H1*A*⋯I1	0.846 (19)	2.78 (2)	3.5761 (16)	157.2 (16)
N1—H1*B*⋯I1^ii^	0.94 (2)	2.77 (2)	3.6156 (16)	150.1 (16)
N4—H4⋯I1^iii^	0.83 (1)	3.15 (2)	3.8882 (14)	149 (2)

Symmetry codes: (i) 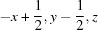; (ii) 
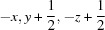
; (iii) 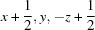.

**Fig. 1.**
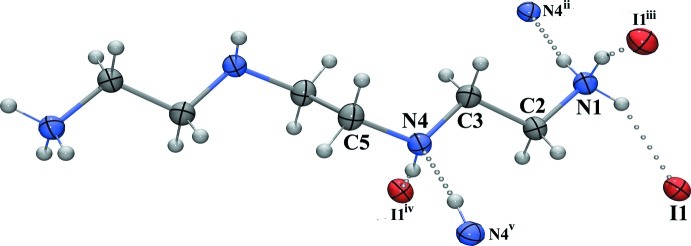
Molecular structure of the title compound showing the atom labeling scheme and 50° probability displacement ellipsoids. Broken lines indicate the N—H···N hydrogen bond and the weak N—H···I intermolecular interactions. Symmetry codes: (ii) -x + 1/2, y - 1/2, z; (iii) -x, y + 1/2, -z + 1/2; (iv) x + 1/2, y, -z + 1/2; (v) -x + 1/2, y + 1/2, z.

**Fig. 2.**
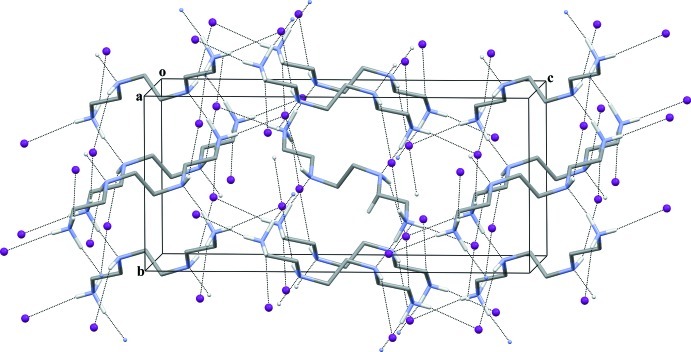
Packing diagram of the title compound viewed offset from along the a axis. Dashed lines indicate N—H···N hydrogen bonds and weak N—H···I intermolecular interactions.forming a three-dimensional network

## supplementary crystallographic information

### Comment

The title compound [C_6_H_20_N_4_^2+^ 2(I^-^)] (1) was obtained during an attempt to prepare an iodide salt of a singly protonated *N*,*N*'-di(2-aminoethyl)-2-aminoethane-1-ammonium ion (C_6_H_19_N_4_^+^ I^-^). In the crystal structure of 1 the cation lies across a centre of inversion with the centre of inversion bisecting the C5—C5^i^ (symmetry code (i): -*x* + 1,-*y* + 1,-*z* + 1) bond. The backbone of the cation does not form a uniformly *trans* chain (Fig. 1): the torsion angles N1—C2—C3—N4 and C2—C3—N4—C5 are -177.22 (14) and 177.58 (13)° respectively, but the C3—N4—C5—C5^i^ torsion angle is 71.5 (2)° (the N4—C5—C5^i^—N4^i^ torsion is, perforce, 180°). Two H atoms of the ammonium group N1, H1A and H1B, form weak intermolecular interactions with two I^-^ anions whereas the third, H1C, is hydrogen bonded to a secondary amine, N4, of an adjacent cation. The refined N1—H1C bond length (1.10 (2) Å) is longer than expected and may indicate a weakening of the N1—H1C bond and a shift of the electron density maximum away from N1 towards the hydrogen bond. There is a weak interaction between the secondary amine N4— H4 donor and a third I^-^ cation. Thus each I^-^ anion is an acceptor for three hydrogen bonds. The hydrogen bonds link the anions and cations in a three-dimensional network (Fig. 2).

The crystal structures of a dihydrate of 1 [C_6_H_20_N_4_^2+^ 2(I^-^) 2(H_2_O)], together with the isostructural Cl^-^ and Br^-^ analogues, have been reported (Ilioudis, *et al.*, 2000). In these structures the C_6_H_20_N_4_^2+^ cations again lie across centres of inversion, but they form uniformly *trans* chains with the magnitudes of the backbone torsion angles all in the range 177.0 (2) – 180°. The presence of water in these hydrates changes the hydrogen bonding / intermolecular interaction pattern compared with 1. Two ammonium and the secondary amine H atoms interact with halide anions as in 1, but there is no direct hydrogen bond between the ammonium group and the secondary amine of a neighbouring cation. Instead, the water molecule acts as an acceptor for a hydrogen bond involving the third ammonium H atom and, in turn, acts as a donor to the secondary amine N atom of a second cation. The second H atom of the water molecule forms a fourth hydrogen bond to a halide anion.

The "kink" in the backbone chain of the cation in 1 probably occurs to accommodate the direct hydrogen bond between the ammonium group and the secondary amine in an adjacent cation, whereas in the hydrates the intervening water molecule in the hydrogen bonding network allows more flexibility and allows the cations to adopt a uniformly *trans* configuration.

### Experimental

0.9 ml of a 57% aqueous solution of HI (6.8 mmol) was added to 1.0 ml of a 6.65*M* aqueous solution of *N*,*N*'-di(2-aminoethyl)-ethane-1,2-diamine (C_6_H_18_N_4_, 6.65 mmol) (QinHuangDao JinLei Chemical Co.Ltd). An exothermic reaction occurred and a greenish-yellow solution was observed. 0.3 ml of water was then added. This solution was heated at 70 °C for 4 h, cooled to room temperature and then left covered for six days. It was then allowed to slowly evaporate by covering the container with perforated aluminium foil. Colourless crystals were obtained after three days of slow evaporation.

### Refinement

H1A, H1B, H1C and H4 were located by a difference map and their coordinates were freely refined, except that the N4—H4 bond length was restrained (target: 0.91 (2) Å, refined length: 0.833 (14) Å) as unrestrained refinement led to an unacceptably short N—H bond length. All of the remaining H atoms were placed in their calculated positions and then refined using the riding model with Atom—H lengths of 0.95 Å, (CH) or 0.99 Å (CH_2_). Isotropic displacement parameters for all hydrogen atoms were set to 1.20 times *U*_eq_ of the parent atom.

### Crystal data

**Table:**

C_6_H_20_N_4_^2+^·2I^−^	*F*(000) = 760
*M**_r_* = 402.06	*D*_x_ = 2.015 Mg m^−^^3^
Orthorhombic, *P**b**c**a*	Mo *K*α radiation, λ = 0.71070 Å
Hall symbol: -P 2ac 2ab	Cell parameters from 8314 reflections
*a* = 8.1253 (2) Å	θ = 1.0–33.7°
*b* = 8.6138 (2) Å	µ = 4.71 mm^−^^1^
*c* = 18.9368 (4) Å	*T* = 180 K
*V* = 1325.38 (5) Å^3^	Block, colourless
*Z* = 4	0.14 × 0.10 × 0.07 mm

### Data collection

**Table:**

Nonius KappaCCD diffractometer	2635 independent reflections
Radiation source: fine-focus sealed tube	1905 reflections with *I* > 2σ(*I*)
Graphite monochromator	*R*_int_ = 0.042
Thin slice ω and φ scans	θ_max_ = 33.7°, θ_min_ = 3.6°
Absorption correction: multi-scan (*SORTAV*; Blessing, 1995)	*h* = −12→12
*T*_min_ = 0.688, *T*_max_ = 0.820	*k* = −13→13
13267 measured reflections	*l* = −29→29

### Refinement

**Table:**

Refinement on *F*^2^	Primary atom site location: structure-invariant direct methods
Least-squares matrix: full	Secondary atom site location: difference Fourier map
*R*[*F*^2^ > 2σ(*F*^2^)] = 0.023	Hydrogen site location: difference Fourier map
*wR*(*F*^2^) = 0.053	H atoms treated by a mixture of independent and constrained refinement
*S* = 0.97	*w* = 1/[σ^2^(*F*_o_^2^) + (0.0251*P*)^2^] where *P* = (*F*_o_^2^ + 2*F*_c_^2^)/3
2635 reflections	(Δ/σ)_max_ = 0.002
67 parameters	Δρ_max_ = 0.55 e Å^−^^3^
1 restraint	Δρ_min_ = −1.05 e Å^−^^3^
0 constraints	

### Special details

**Table:**

Geometry. All e.s.d.'s (except the e.s.d. in the dihedral angle between two l.s. planes) are estimated using the full covariance matrix. The cell e.s.d.'s are taken into account individually in the estimation of e.s.d.'s in distances, angles and torsion angles; correlations between e.s.d.'s in cell parameters are only used when they are defined by crystal symmetry. An approximate (isotropic) treatment of cell e.s.d.'s is used for estimating e.s.d.'s involving l.s. planes.
Refinement. Refinement of *F*^2^ against ALL reflections. The weighted *R*-factor *wR* and goodness of fit *S* are based on *F*^2^, conventional *R*-factors *R* are based on *F*, with *F* set to zero for negative *F*^2^. The threshold expression of *F*^2^ > σ(*F*^2^) is used only for calculating *R*-factors(gt) *etc*. and is not relevant to the choice of reflections for refinement. *R*-factors based on *F*^2^ are statistically about twice as large as those based on *F*, and *R*- factors based on ALL data will be even larger.

### Fractional atomic coordinates and isotropic or equivalent isotropic displacement parameters (Å^2^)

**Table:**

	*x*	*y*	*z*	*U*_iso_*/*U*_eq_	
I1	0.151716 (14)	0.140690 (14)	0.149443 (6)	0.03028 (5)	
N1	0.07593 (17)	0.28497 (19)	0.32351 (8)	0.0259 (3)	
H1A	0.063 (2)	0.258 (2)	0.2810 (11)	0.031*	
H1B	−0.012 (3)	0.349 (2)	0.3373 (10)	0.031*	
H1C	0.093 (3)	0.175 (3)	0.3520 (9)	0.031*	
C2	0.2280 (2)	0.37794 (18)	0.32818 (10)	0.0273 (4)	
H2A	0.2138	0.4755	0.3012	0.033*	
H2B	0.3201	0.3194	0.3067	0.033*	
C3	0.2694 (2)	0.4159 (2)	0.40401 (8)	0.0282 (3)	
H3A	0.1759	0.4707	0.4264	0.034*	
H3B	0.2901	0.3190	0.4307	0.034*	
N4	0.41655 (16)	0.51470 (17)	0.40564 (7)	0.0241 (3)	
H4	0.4916 (18)	0.465 (2)	0.3862 (10)	0.029*	
C5	0.4643 (2)	0.5645 (2)	0.47735 (8)	0.0277 (3)	
H5A	0.3663	0.6076	0.5014	0.033*	
H5B	0.5467	0.6488	0.4734	0.033*	

### Atomic displacement parameters (Å^2^)

**Table:**

	*U*^11^	*U*^22^	*U*^33^	*U*^12^	*U*^13^	*U*^23^
I1	0.02627 (7)	0.02885 (7)	0.03570 (8)	−0.00126 (4)	0.00130 (4)	0.00008 (4)
N1	0.0236 (7)	0.0278 (8)	0.0264 (7)	−0.0015 (6)	−0.0020 (6)	0.0004 (6)
C2	0.0240 (8)	0.0330 (10)	0.0249 (8)	−0.0035 (7)	−0.0018 (6)	0.0000 (6)
C3	0.0270 (8)	0.0331 (9)	0.0245 (8)	−0.0044 (7)	−0.0020 (6)	0.0020 (6)
N4	0.0219 (6)	0.0265 (7)	0.0238 (6)	−0.0007 (5)	−0.0013 (5)	−0.0011 (5)
C5	0.0305 (8)	0.0253 (9)	0.0271 (8)	0.0005 (7)	−0.0045 (7)	−0.0004 (6)

### Geometric parameters (Å, º)

**Table:**

N1—C2	1.475 (2)	C3—H3A	0.9900
N1—H1A	0.846 (19)	C3—H3B	0.9900
N1—H1B	0.94 (2)	N4—C5	1.476 (2)
N1—H1C	1.10 (2)	N4—H4	0.833 (14)
C2—C3	1.511 (2)	C5—C5^i^	1.519 (3)
C2—H2A	0.9900	C5—H5A	0.9900
C2—H2B	0.9900	C5—H5B	0.9900
C3—N4	1.467 (2)		
			
C2—N1—H1A	108.2 (13)	C2—C3—H3A	109.9
C2—N1—H1B	107.8 (12)	N4—C3—H3B	109.9
H1A—N1—H1B	109.4 (17)	C2—C3—H3B	109.9
C2—N1—H1C	109.6 (13)	H3A—C3—H3B	108.3
H1A—N1—H1C	104.0 (16)	C3—N4—C5	113.71 (12)
H1B—N1—H1C	117.5 (16)	C3—N4—H4	106.6 (13)
N1—C2—C3	111.17 (15)	C5—N4—H4	111.4 (13)
N1—C2—H2A	109.4	N4—C5—C5^i^	114.02 (18)
C3—C2—H2A	109.4	N4—C5—H5A	108.7
N1—C2—H2B	109.4	C5^i^—C5—H5A	108.7
C3—C2—H2B	109.4	N4—C5—H5B	108.7
H2A—C2—H2B	108.0	C5^i^—C5—H5B	108.7
N4—C3—C2	109.09 (13)	H5A—C5—H5B	107.6
N4—C3—H3A	109.9		
			
N1—C2—C3—N4	−177.22 (14)	C3—N4—C5—C5^i^	71.5 (2)
C2—C3—N4—C5	177.58 (13)		

Symmetry code: (i) −*x*+1, −*y*+1, −*z*+1.

### Hydrogen-bond geometry (Å, º)

**Table:**

*D*—H···*A*	*D*—H	H···*A*	*D*···*A*	*D*—H···*A*
N1—H1*C*···N4^ii^	1.10 (2)	1.71 (2)	2.800 (2)	168.3 (19)
N1—H1*A*···I1	0.846 (19)	2.78 (2)	3.5761 (16)	157.2 (16)
N1—H1*B*···I1^iii^	0.94 (2)	2.77 (2)	3.6156 (16)	150.1 (16)
N4—H4···I1^iv^	0.83 (1)	3.15 (2)	3.8882 (14)	149 (2)

Symmetry codes: (ii) −*x*+1/2, *y*−1/2, *z*; (iii) −*x*, *y*+1/2, −*z*+1/2; (iv) *x*+1/2, *y*, −*z*+1/2.
